# Bioactivity and Regenerative Potential of Cannabidiol in Human Dental Pulp Stem Cells: A Scoping Review of In Vitro Studies

**DOI:** 10.1155/tswj/6756387

**Published:** 2026-02-26

**Authors:** Lorena Gomes Guimarães, Wesley Viana de Sousa, Silmara de Andrade Silva, Christianne Velozo, Carolina Viana Vasco Lyra, Larissa Sousa Rangel, Maria Alice Lopes Pereira, Diana Albuquerque

**Affiliations:** ^1^ Department of Restorative Dentistry and Endodontics, Dental College of Pernambuco, University of Pernambuco, Recife, Pernambuco, Brazil, ufpe.br

**Keywords:** cannabidiol, cell differentiation, dental pulp, endodontics, stem cells

## Abstract

**Introduction:**

Cannabidiol (CBD), a nonpsychoactive compound derived from *Cannabis sativa*, has shown potential to influence cellular processes that are important for dental tissue repair. The aim of this scoping review was to map in vitro studies evaluating the influence of CBD on the osteogenic/odontogenic differentiation of human dental pulp stem cells (hDPSCs) in order to contribute to a better understanding of its therapeutic potential.

**Methods:**

The review followed the Arksey and O′Malley framework, supported by the JBI Manual and PRISMA‐ScR guidelines. The protocol was registered on OSF (osf.io/zfhca/). Comprehensive searches were conducted from January to June 2025 in PubMed, EMBASE, BVS, Scopus, Web of Science, ScienceDirect, and SciELO. Only studies published in English were included.

**Results:**

Thirty articles were identified, and three in vitro studies met the eligibility criteria. At low concentrations (0.1–5 *μ*M), CBD improved hDPSC viability, proliferation, migration, and differentiation. CBD also activated the mitogen‐activated protein kinase (MAPK) and wingless‐related integration site/beta‐catenin signaling (WNT/*β*‐catenin) pathways and increased the expression of odontogenic markers such as Sialophosphoprotein (DSPP), Runt‐related transcription Factor 2 (RUNX2), and osteocalcin.

**Conclusion:**

CBD shows promise as a bioactive molecule in regenerative endodontics, supporting mineralization, regulating inflammatory mediators, and promoting critical cellular activities in hDPSCs. Nevertheless, the available evidence is limited and further preclinical and clinical studies are essential to develop therapeutic protocols and assess long‐term safety. These preliminary findings indicate CBD as a novel candidate for regenerative strategies in endodontics.

## 1. Introduction

The dental pulp, which is essential for dentin‐pulp homeostasis, is uniquely enclosed in mineralized tissue, a fact that limits vascularization and cell migration. This structural constraint increases the pulp′s susceptibility to bacterial invasion and tissue damage [1, 2]. Preserving pulp vitality in cases of reversible pulpitis with exposure due to caries, trauma, or iatrogenesis remains a key clinical challenge [3]. Unlike irreversible pulpitis that requires root canal treatment, the successful treatment of reversible pulpitis relies on eliminating inflammation to promote tissue repair [4]. Within this context, understanding the modulatory bioactivity of emerging compounds such as cannabidiol (CBD) in dental pulp stem cells (DPSCs) is essential for developing biologically driven therapeutic strategies.

Vital pulp therapy seeks to maintain or restore the dentin‐pulp complex by applying bioactive materials that stimulate odontogenesis [5, 6]. Calcium hydroxide has been widely used due to its alkaline pH and calcium ion release, promoting reparative bridge formation. However, the low adhesion and potential cytotoxicity of calcium hydroxide, associated with the formation of porous bridges, limit its performance [7]. Newer materials such as mineral trioxide aggregate, calcium‐enriched mixture cement, and Biodentine have shown promising results, although they still fall short in reproducing true regenerative, anti‐inflammatory, or analgesic effects since the dentin formed differs from that produced during natural dentinogenesis [3].

Tissue regeneration relies on the recruitment, proliferation, and differentiation of DPSCs into odontoblast‐like cells, which secrete extracellular matrix and form reparative dentin [8–10]. As mesenchymal stromal cells, DPSCs exhibit immunomodulatory activity and low immunogenicity, properties that render them suitable for clinical use [11, 12]. However, inflammatory mediators, especially tumor necrosis factor alpha (TNF‐*α*), can modulate hDPSC viability and mineralization potential in both positive and negative ways. Positively, TNF‐*α* acting through the TNF‐*α*/TNFR1 axis has been associated with reparative dentin formation following pulp‐capping procedures in vivo. Conversely, increased mRNA expression of TNF‐*α*, IL‐6, and IL‐1*β* negatively affects human dental pulp stem cells (hDPSCs) by inhibiting matrix mineralization and downregulating osteopontin, Type I collagen, alkaline phosphatase, and runt‐related transcription Factor 2 (RUNX2) [11, 13–16]. These findings reinforce the need to explore agents capable of supporting the function of DPSCs under inflammatory conditions.

Among such agents, *Cannabis sativa* has attracted attention due to its analgesic, chemotactic, and regenerative properties [17, 18]. CBD, its nonpsychoactive component, has shown promise in managing inflammation and promoting tissue repair in various medical fields [17, 19]. These effects are mediated by the endocannabinoid system through cannabinoid Type 1 (CB1) and cannabinoid Type 2 (CB2) receptors, which regulate immune responses, neuroprotection, and homeostasis [20, 21]. Studies have shown that CB1 and CB2 receptors are expressed on DPSCs and are involved in reparative dentinogenesis, including calcium influx modulation [11, 22, 23]. Therefore, the aim of this scoping review was to map in vitro studies evaluating the influence of CBD on the osteogenic/odontogenic differentiation of hDPSCs in order to contribute to a better understanding of its therapeutic potential.

## 2. Materials and Methods

This scoping review was conducted in accordance with the five‐stage framework proposed by Arksey and O′Malley [24]: identifying the research question; identifying relevant studies; selecting the studies; charting the data, and collating, summarizing, and reporting the results. The research question was “What is the influence of cannabidiol on the activity of dental pulp stem cells?” To standardize this review, the JBI Manual for Evidence Synthesis [25] and the Preferred Reporting Items for Systematic Reviews and Meta‐Analyses extension for Scoping Reviews (PRISMA‐ScR) checklist [26] were also followed. The methods were registered on the Open Science Framework (osf.io/zfhca).

### 2.1. Eligibility Criteria

**Figure 1:**
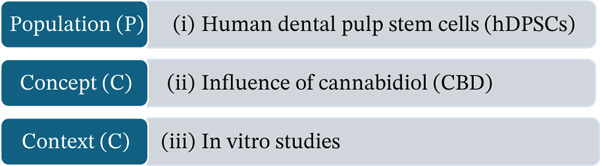
PCC strategy of the included article.

This review was structured using the population, concept, and context (PCC) framework [24] (Figure 1). A comprehensive search was performed between January and June 2025. Studies that met the following eligibility criteria were selected: in vitro studies that investigated the effects of CBD on DPSCs, including its mechanisms of action and potential application in regenerative therapies based on osteogenic/odontogenic differentiation.

### 2.2. Information Sources

The studies retrieved through the search strategy were exported in CIW/RIS format from the databases to the Rayyan QCRI online platform (https://www.rayyan.ai) (RRID:SCR_017584) for the removal of duplicate records [27]. All authors contributed to the development of the search strategy. Two independent groups of reviewers conducted the electronic search across the following databases: PubMed, EMBASE, BVS, Scopus, Web of Science, ScienceDirect, and SciELO.

### 2.3. Search Strategy

For the search, descriptors and Boolean operators were combined as follows: 1# (“Endodontics” OR “Regenerative Endodontics” OR “Regenerative Medicine”), 2# (“Cannabinoid” OR “Cannabis” OR “Cannabidiol”), and 3# (“Human Dental Pulp Cell” OR “Stem Cell Research”). The search strategy applied #1 AND #2 AND #3, with publication period restriction of 5 years (Table 1).

**Table 1:** Database search strategies for cannabidiol (CBD) and dental pulp stem cell studies.

Database	Search Terms	Boolean operators
PubMed/MEDLINE	(((((Endodontics) OR (Regenerative Endodontics)) OR (Regenerative Medicine)))) AND (((Cannabinoid) OR (Cannabis)) OR (Cannabidiol)))) AND ((Human Dental Pulp Cell) OR (Stem Cell Research) AND (y_5[Filter]))	AND, OR
EMBASE	(“endodontics” OR “regenerative endodontics” OR “regenerative medicine”) AND (“cannabinoid” OR “cannabis” OR “cannabidiol”) AND (“human dental pulp cell” OR “stem cell research”) AND [2019–2024]/py	AND, OR
BVS (LILACS/BIREME)	(Endodontics OR “Regenerative Endodontics” OR “Regenerative Medicine”) AND (Cannabinoid OR Cannabis OR Cannabidiol) AND (“Human Dental Pulp Cell” OR “Stem Cell Research”) AND (year_cluster:(“2020” OR “2021” OR “2022” OR “2023” OR “2024”))	AND, OR
Scopus	TITLE‐ABS‐KEY (“Endodontics” OR “Regenerative Endodontics” OR “Regenerative Medicine”) AND TITLE‐ABS‐KEY (“Cannabinoid” OR “Cannabis” OR “Cannabidiol”) AND TITLE‐ABS‐KEY (“Human Dental Pulp Cell” OR “Stem Cell Research”) AND PUBYEAR >2018	AND, OR
Web of Science	(TS = (“Endodontics” OR “Regenerative Endodontics” OR “Regenerative Medicine”)) AND (TS = (“Cannabinoid” OR “Cannabis” OR “Cannabidiol”)) AND (TS = (“Human Dental Pulp Cell” OR “Stem Cell Research”)) AND PY = (2019–2024)	AND, OR
ScienceDirect	(“Endodontics” OR “Regenerative Endodontics” OR “Regenerative Medicine”) AND (“Cannabinoid” OR “Cannabis” OR “Cannabidiol”) AND (“Human Dental Pulp Cell” OR “Stem Cell Research”) Filters: 2019–2024; Review articles; Research articles; Other	AND, OR
SciELO	1# (Endodontics) OR (Regenerative Endodontics) OR (Regenerative Medicine)2# (Cannabinoid) OR (Cannabis) OR (Cannabidiol)3# (Human Dental Pulp Cell) OR (Stem Cell Research)4# #1 AND #2 AND #3	AND, OR

As recommended by the PRISMA statement (3), the studies were selected by two independent groups (G). Therefore, the authors split up into: G1 Wesley Viana de Sousa (W.V.S.) and Lorena Gomes Guimarães (L.G.G.) and G2 Christianne Velozo (C.V.) and Silmara de Andrade Silva (S.A.S.). Each group performed the screening in parallel. Diana Santana de Albuquerque (D.A.) assisted in resolving disagreements and refining eligibility criteria, and supervised the entire process and ensured methodological consistency throughout the review. Reviewer agreement was assessed by calculating Kappa scores [28]. Discrepancies were resolved through discussion with the final reviewer (D.A.).

### 2.4. Assessment of Evidence Quality of the Included Studies

**Figure 2:**
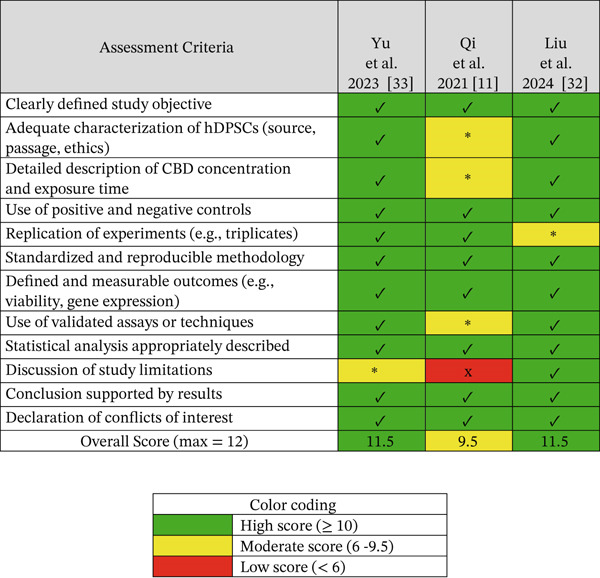
Quality assessment based on a customized 12‐item checklist adapted from QUADAS, ToxRTool, and OHAT. Symbols: ✓ = *criterion fully met* (1 point), ∗ = *partially met* (0.5), and ✘ = *not met* (0). CBD, cannabidiol; hDPSCs, human dental pulp stem cells. Overall, two studies (Yu et al. [11] and Liu et al. [32]) demonstrated high methodological quality (11.5/12), whereas Qi et al. 2021 showed moderate quality (9.5/12).

Out of the 30 studies identified, three were included in the research. These three articles were assessed for methodological quality using a 12‐item checklist adapted from QUADAS, ToxRTool, and OHAT, composed of the following items: (1) clearly defined study objective, (2) adequate characterization of hDPSCs (source, passage, and ethics), (3) detailed description of CBD concentration and exposure time, (4) use of positive and negative controls, (5) replication of experiments (e.g., triplicates), (6) standardized and reproducible methodology, (7) defined and measurable outcomes (e.g., viability and gene expression), (8) use of validated assays or techniques, (9) statistical analysis appropriately described, (10) discussion of study limitations, (11) conclusion supported by results, and (12) declaration of conflicts of interest [29–31]. Out of the three articles included in the research, two of them [11, 32] achieved the highest scores, whereas the study of Qi et al. [33] scored slightly lower, indicating moderate to high methodological rigor of the included studies. The complete results are presented in Figure 2.

### 2.5. Evidence Synthesis

The following data were extracted from the in vitro studies: first author and year of publication, aim, experimental methodology, outcomes, results, and conclusions. Each group (Group 1: W.V.S., L.G.G.; Group 2: C.V., S.A.S.) extracted and reviewed the information, and the findings were discussed in a consensus meeting with a coordinating author (D.A). The extracted data were subsequently mapped to support the framework of this scoping review [25].

### 2.6. Additional Analysis

The interexaminer agreement was assessed using Cohen′s kappa (*κ*) coefficient during the study selection process, encompassing both the title/abstract screening and the full‐text evaluation phases. The obtained *κ* values and their corresponding 95% confidence intervals are reported in the Results section.

## 3. Results

**Figure 3:**
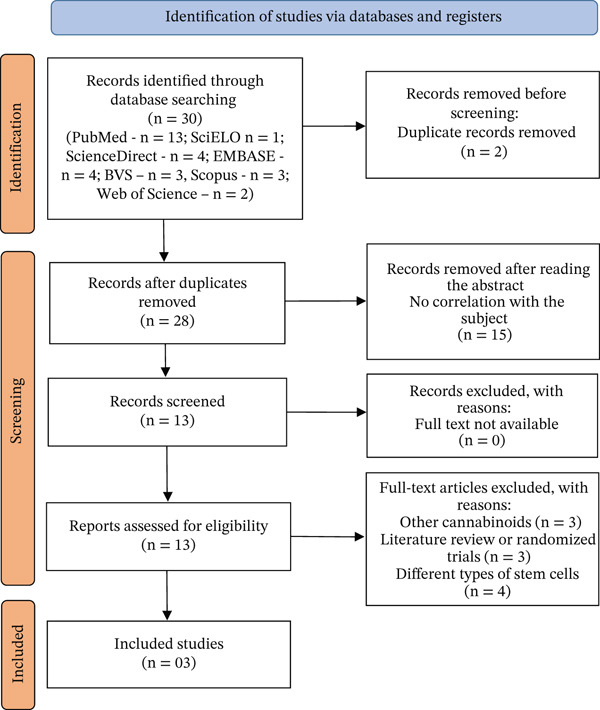
PRISMA‐ScR flowchart visually represents how records were retrieved across databases, screened, excluded, and ultimately included in the final synthesis.

**Figure 4:**
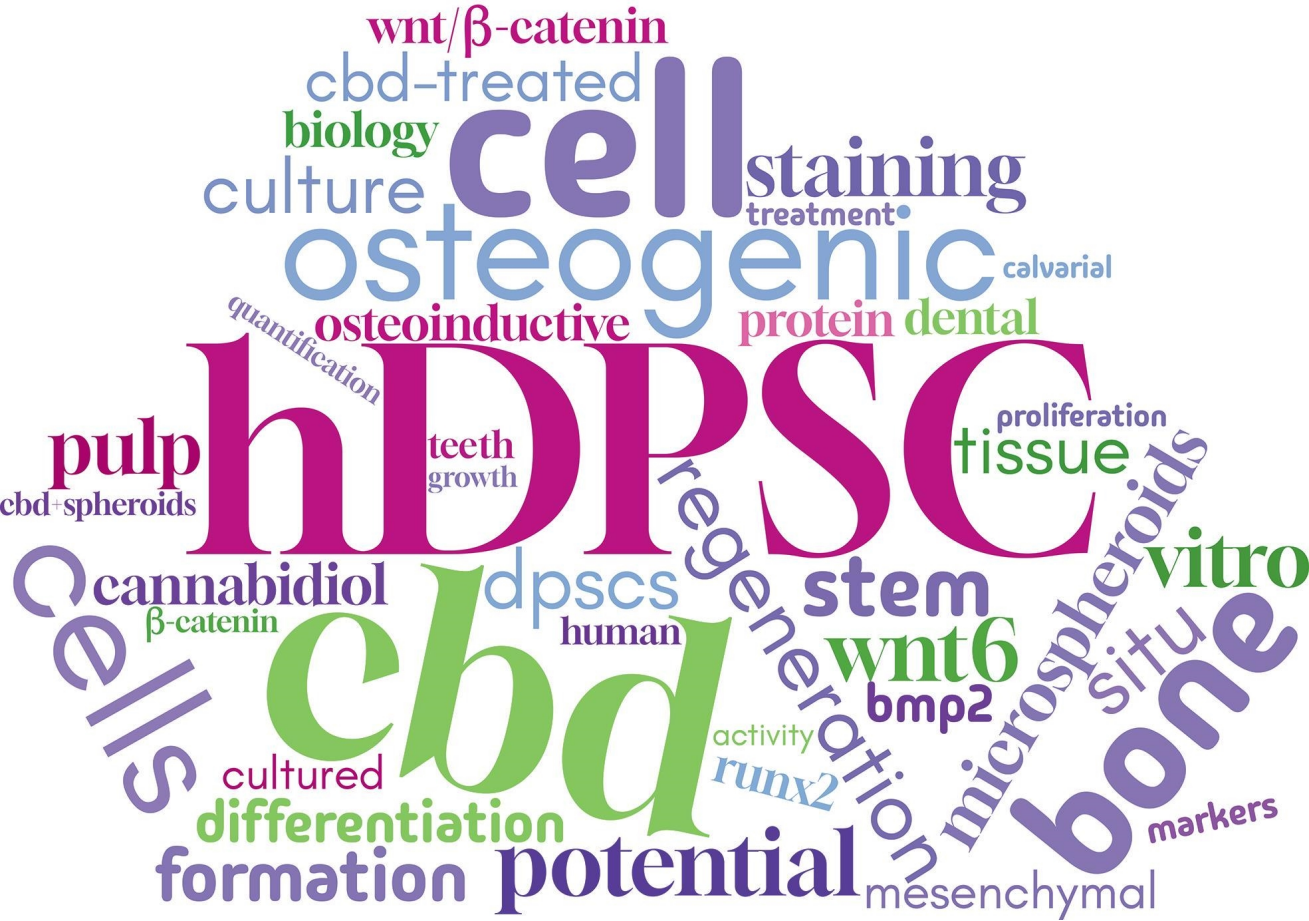
Word cloud representing the articles included. The more frequently the word was mentioned across the included articles, the bigger it appears in the word cloud.

The database search identified 30 articles. During the eligibility screening process, duplicate records (*n* = 2) and articles unrelated to the research topic (*n* = 15) were removed. Of the remaining articles, 13 had their full text read and three were included in the review. The identification of the studies is described in Figure 3. The studies that were not included in the final scoping review (*n* = 25) are listed in Table 2, along with their respective reasons for exclusion. Table 3 shows the synthesis of the analyzed articles. Table 4 presents a comparative synthesis of the main materials and methods used in the selected studies. A visual representation of the most frequent terms extracted from the included studies is shown in the word cloud in Figure 4, created using the website https://www.wordclouds.com/, which highlights the core concepts related to CBD bioactivity in hDPSCs.

**Table 2:** Excluded studies on cannabidiol (CBD) and dental pulp stem cell research.

	Author and year	Reason for exclusion
1	Altieri et al. 2023	Research that portrays atrial remodeling does not mention issues related to revision.
2	Arceri et al. 2023	Review of the literature on the ECS in the renal system.
3	Chandy et al. 2024	It only deals with adverse effects of *Cannabis sativa* regarding recreational use, in addition to being a review.
4	Chandy et al. 2022	It is a review that defines only the possible impacts of environmental factors on the body from the use of stem cells.
5	Chang et al. 2022	It refers only to the impact of basic fibroblast growth factor on apical papilla stem cells. It does not mention CBD.
6	Greco et al. 2020	The article focuses only on amniotic epithelial cells
7	Hosseinkhani et al. 2023	Review of literature that deals only with gene therapy.
8	Jain et al. 2021	It only exposes about maternal immune activation.
9	Jiang et al. 2021	The article, despite portraying the application of CBD, does so in a mouse with traumatic brain injury.
10	Lam et al. 2022	It defines the neurophysiological role of Collagen VI, and therefore does not align with the topic.
11	Lee et al. 2021	Article presents only the action of the endocannabinoid system in atrial remodeling.
12	López‐Tofiño et al. 2023	It deals with the impact of substances on gastrointestinal disorders, having no relation to research.
13	López‐Tofiño et al. 2024	It is not related to the theme because it deals with drugs and their activity on gastrointestinal motility
14	López‐Tofiño et al. 2024	Outside the scope of the research because it portrays the activity of drugs in rats.
15	Marques Azzini et al. 2023	This is a literature review on CBD in chronic musculoskeletal pain.
16	Mesas et al. 2025	Despite reporting the effects of cannabidiol on stem cells, it is not classified for the research because it is a systematic review
17	Miller et al. 2021	The article reports an in vitro experiment, as well as in animals and with other stem cell lines.
18	Peng; Year and Tran 2024	It comprises only gum stem cells.
19	Ruhl et al. 2021	Study portrays only the action of endocannabinoids.
20	Salwa and Kumar 2021	It deals with the prospects of treating Alzheimer′s with stem cells, but does not allude to cannabinoids.
21	Silva et al. 2025	Double‐blind clinical trial study of oral cannabidiol‐rich cannabis
22	Tomer et al. 2022	It deals with the impact of CBD on macrophages; it is not about stem cells or differentiation.
23	Vera et al. 2024	In addition to being a literature review, its theme deals with the neurotoxic effects of chemotherapy on the enteric nervous system.
24	Verdikt et al. 2024	Reports the behavior of germ cells after recreational use of *Cannabis sativa*.
25	Wang et al. 2025	It has no relation to the research because it portrays the effect of storing breast milk on lipids.

Abbreviations: CBD, cannabidiol; ECS, endocannabinoid system.

**Table 3:** Synthesis of included studies on cannabidiol (CBD) and dental pulp stem cells.

First author/year	Aim	Experimental methodology	Outcomes	Results	Conclusion
Yu et al. 2023 [33]	To analyze the effect of CBD on DPSC‐mediated pulp regeneration under normal and inflammatory conditions	Culture of hDPSCs with CBD at different concentrations with and without TNF‐*α*; assays for viability, migration, differentiation, and gene and protein expression	Viability, migration, differentiation, angiogenesis, marker expression, and inflammatory modulation	CBD increased viability and differentiation at low doses and reduced TNF‐*α*‐induced inflammation	CBD is promising for pulp regeneration in DPSCs and inflammation modulation.
Liu et al. 2024 [32]	To assess whether CBD enhances the osteogenic potential of DPSC‐derived microspheroids	Formation of microspheroids with CBD‐treated DPSCs; in vitro evaluation and implantation in cranial bone defects in mice	Mineralization, WNT6/*β*‐catenin expression, in vivo bone regeneration	CBD enhanced osteogenesis and bone regeneration via WNT6/*β*‐catenin pathway	CBD amplifies osteogenic potential and may benefit bone tissue engineering.
Qi et al. 2021 [11]	To explore the osteo/odontogenic potential of CBD focusing on angiogenic factors	Western blot, PCR, migration assays, VEGF and ICAM‐1 expression analyses	Angiogenesis, osteogenic differentiation, and expression of vascular and osteogenic markers	CBD‐induced VEGF and ICAM‐1 expression along with classical osteogenic markers	CBD is promising in pulp regeneration through differentiation and angiogenesis.

Abbreviations: CB1, cannabinoid Type 1; CB2, cannabinoid receptor Type 2; CBD, cannabidiol; DPSCs, dental pulp stem cells; hDPSCs, human dental pulp stem cells; ICAM‐1, intercellular adhesion Molecule‐1; MAPK, mitogen‐activated protein kinase; PCR, polymerase chain reaction; TNF‐*α*, tumor necrosis factor alpha; VEGF, vascular endothelial growth factor; WNT6, wingless‐type MMTV integration site family Member 6.

**Table 4:** Comparative synthesis of the materials and methods used in the included studies. This table contains the main information on the materials and methods used in the selected studies. It is divided into three parts: (1) information about hDPSCs (donor age, quantity of donors, number of teeth, oral health, tooth type, purpose of extraction, hDPSCs, culture medium, passage number, and others); (2) information about CBD (source of CBD, purity of CBD, preparation of CBD, and dose range and duration), and (3) information about the tests (reason for the test, name of the test, duration/assay time points, quantity of well plates, and density).

		Qi et al. 2021 [33]	Yu et al. 2023 [11]	Liu et al. 2024 [32]
**Informations about the hDPSCs**	Donor age	—	12–20 years	18–25 years
	Quantity of donors	—	20 patients	20 patients (10 male, 10 female, 1–2 tooth/patient)
	Number of teeth	—	—	30 teeth
	Oral health	—	—	Patients without caries, periodontal disease, periapical lesion, and systemic inflammatory diseases.
	Tooth type	Premolars	Premolars and wisdom teeth	Premolars
	Purpose of extraction	Orthodontic purposes	Orthodontic purposes	Orthodontic purposes
	hDPSCs culture medium	*α*‐MEM (Hyclone, Logan, Utah, United States) supplemented with 20% fetal bovine serum (FBS, Hyclone) and 1% penicillin/streptomycin	*α*‐MEM (Gibco, Waltham, Massachusetts, United States), 15% FBS, (Gibco, Waltham, Massachusetts, United States), and 100 U/mL penicillin/streptomycin (Gibco, Waltham, Massachusetts, United States) at 37°C in a 5% CO_2_ incubator. The medium was refreshed every 3 days.	*α*‐MEM (Gibco, United States) containing 15% FBS (Gibco, United States) and 1% penicillin/streptomycin, and cultured at 37°C in an environment with 5% CO_2_ with the medium replacement every 3 days
	Passage number	hDPSCs at passages 3–5 (P3–P5)	hDPSCs at passages 3–5 (P3–P5)	hDPSCs at passages 2–5 (P2–P5)
	Others			Development of microspheroids and characterization: DPSC were trypsinized, and 5 × 10^5^ cells were diluted in 500 *μ*L of serum‐free chemically defined medium in 24‐well plates. Approximately 250 cells migrated in each well and self‐aggregated to form microspheroid.

**Informations about the CBD**	Source of CBD	Sigma (St. Louis, Missouri)	—	Sigma (St. Louis, Missouri)
	Purity of CBD	≥ 98%	—	≥ 98%
	Preparation of CBD	—	—	—
	Dose range and duration	0.1, 0.5, 1, 5, 10, 50, and 100 *μ*M for maximum 21 days	0, 0.1, 0.5, 2.5, and 12.5 *μ*M for maximum 28 days	0, 0,1, 0,5, 2,5 e and 12.5 *μ*M for maximum 28 days

**Informations about the tests**(Name of the test; duration/assay; time points; quantity of well plates; and density)	Cell proliferation assay	MTT: 24, 48, and 72 h – 96‐well plates – 5000 cells/well in 200 *μ*L	Cell Counting Kit‐8 (CCK‐8) assay – 96‐well plates – 3 × 10^3^ cells/well	CCK‐8 assay – 96‐well plates – 2 × 10^3^ cells/well
	Analysis of DPSC surface markers	—	Flow cytometry of hDPSC surface markers (D34, CD45, CD44, CD73, CD90, and CD105)	Flow cytometry of DPSC surface markers (CD29, CD34, CD44, CD45, CD73, CD90, CD105, and IgG1)
	Odonto/osteogenic differentiation analyses	Odonto/osteogenic differentiation – 21 days – 12‐well plates	Alizarin red staining: 28 days – 48‐well platesOil red O staining: 28 days – 48‐well platesAlcian blue staining: 21 days – 48‐well plates	Alizarin red staining: 21 days ‐ 6‐well plates – 105 cells/wellOil red O staining: 28 days ‐6‐well plates – 105 cells/wellAlcian blue staining: 21 days – 48‐well plate – 4 × 10^4^ cells
	Assess the osteogenic phenotype	Von Kossa: 7–21 daysWestern blot: 5‐, 15‐, 30‐, and 60‐min	Alkaline phosphatase (ALP) staining and activity: after 4 and 7 days of culture – 48‐well plates – 1.5 × 10^4^ cells/wellAlizarin Red S (ARS) staining: 14 days – 48‐well plates – 1.5 × 10^4^ cells/wellWestern blot assayimmunofluorescence staining: 3 days	ALP staining and activity: after 4 and 7 days of culture ‐ 48‐well culture plates – 2 × 10^4^ cells/wellMatrix mineralization assays: 21 days – 48‐well culture plates – 2 × 10^4^ cells/wellWestern blot assayimmunofluorescence staining: 14 days
	Asses the migration potencial	Scratch assay: 6, 12, 24, and 48 h – 6‐well plates	Scratch wound and healing assay: 24 and 48 h – 6‐well plates	—
	Colony forming	—	—	Colony‐forming unit assay: 7 days – 6‐well plates – 500 cells/well
	Detection of odontogenic markers	Real‐time polymerase chain reaction (RT‐qPCR) assay to detect DSPP, DMP‐1, Runx2, OPN, ALP, VEGFR1 and ICAM‐1, and COL‐I and III	RNA isolation and RT‐qPCR	RT‐qPCR analysis for ALP, BMP2, RUNX2, OPN, OCN, and WNT6

Abbreviations: *α*‐MEM, alpha‐modified minimal essential media; ALP, alkaline phosphatase; BMP2, bone morphogenetic protein 2; CBD, cannabidiol; CCK‐8, Cell Counting Kit‐8; COL‐I and ‐III, collagen Type I and III; DMP‐1, dentin matrix Protein‐1; DPSCs, dental pulp stem cells; DSPP, sialophosphoprotein; FBS, fetal bovine serum; hDPSCs, human dental pulp stem cells; ICAM‐1, intercellular adhesion Molecule‐1; MTT, 3‐[4,5‐dimethylthiazol‐2‐yl]‐2,5 diphenyl tetrazolium bromide; OCN, osteocalcin; OPN, osteopontin; RT‐qPCR (Real‐time polymerase chain reaction); RUNX2, runt‐related transcription Factor 2; VEGFR1, vascular endothelial growth factor Receptor 1; WNT6, wingless‐type MMTV integration site family, Member 6.

Interreviewer agreement was assessed separately for each database during the title/abstract screening phase. Agreement levels ranged from substantial to almost perfect across all sources, with the following kappa (*κ*) values: PubMed (*κ* = 0.83), EMBASE (*κ* = 0.79), BVS (*κ* = 0.76), Scopus (*κ* = 0.81), Web of Science (*κ* = 0.82), ScienceDirect (*κ* = 0.77), and SciELO (*κ* = 0.75). These values collectively indicate *almost perfect agreement* between reviewers.

## 4. Discussion

This scoping review compiled in vitro studies that investigated the effects of CBD on the osteogenic/odontogenic potential of hDPSCs. This research found data indicating that CBD is a substance capable of increasing the viability, migration, proliferation, and differentiation of hDPSCs through the upregulation of osteogenic/odontogenic markers, including in inflammatory environments [11, 32, 33].

There are reports in the literature indicating that CBD is biocompatible with hDPSCs [32, 33]. Three studies assessed cell viability using either the Cell Counting Kit 8 (CCK‐8) or 3‐[4,5‐dimethylthiazol‐2‐yl]‐2,5 diphenyl tetrazolium bromide (MTT) assay, which yielded convergent findings despite methodological differences: lower concentrations of CBD promoted increases in cell viability and proliferation, whereas higher concentrations induced cytotoxicity, characterizing a dose‐dependent biphasic effect. However, the studies diverge regarding the ideal concentration. Qi et al. [33] identified a mitotic peak at 5 *μ*M, whereas Yu et al. [11] suggested 2.5 *μ*M as the optimal concentration using a density of 3 × 10^3^ cells/well, with the onset of cytotoxic effects at 12.5 *μ*M. Liu et al. [32] reported similar findings using 12.5 *μ*M as the highest dose with CCK‐8 and a 2 × 10^3^ cells/well density. Qi et al. [33] tested the highest concentrations and only reported cytotoxic effects at 100 *μ*M, using a MTT assay and a large density of cells (5 × 10^3^ cells/well).

Cell migration, a key event in dentin repair, was also enhanced by CBD at concentrations ranging from 1 to 2.5 *μ*M, as demonstrated in scratch wound assays [11, 33]. These findings suggest that CBD facilitates cell recruitment to the site of injury. Furthermore, hDPSCs exhibited multilineage differentiation potential, including adipogenic, chondrogenic, and osteogenic pathways, confirmed by lineage‐specific staining with Oil Red O, Alcian Blue, and Alizarin Red, respectively, further supporting their cellular plasticity [11, 32].

The included studies also reported the expression of osteogenic/odontogenic markers in hDPSCs treated with CBD [32, 33]. Qi et al. [33] found extracellular matrix formation and induction of biomineralization in these cells. Real‐time polymerase chain reaction (RT‐qPCR) revealed the upregulated expression of Types I and III collagens, major fibrous components of the extracellular matrix in dental pulp and dentin, as well as significant upregulation of odontogenic differentiation‐related genes, including dentin sialophosphoprotein (DSPP), dentin matrix acidic Phosphoprotein 1, alkaline phosphatase, RUNX2, and osteopontin.

Von Kossa staining revealed a significant increase in mineralized area, corroborating the findings of Liu et al. [32] who evaluated osteogenic microspheroids derived from hDPSCs (70 *μ*m in diameter) treated with CBD. These microspheroids exhibited upregulated expression of osteogenic markers such as bone morphogenetic Protein 2 (BMP2), osteocalcin, osteopontin, and RUNX2 compared with untreated control groups. Moreover, similar outcomes were observed for isolated hDPSCs treated with CBD, reinforcing the central hypothesis of this review: CBD, in addition to maintaining cell viability, may positively modulate the osteogenic differentiation and mineralization of hDPSCs, highlighting its potential as a bioactive agent in regenerative strategies.

The study by Qi et al. [33] highlighted that CBD can increase the expression of vascular endothelial growth factor and intercellular adhesion Molecule 1 in treated hDPSCs. These markers are associated with the stimulation of neovascularization and cellular adhesion during the recruitment of endothelial cells; both events are essential for the revascularization of damaged tissues. These findings expand our understanding of the effects of CBD by showing that, in addition to enhancing cell viability and differentiation, CBD may also support pulp tissue vascularization. The latter is critical for the success of regenerative strategies since maintenance of the local microcirculation directly influences the survival of transplanted stem cells, nutrient delivery, and removal of metabolic waste. Therefore, the presence of those markers could indicate a possible potential as a strategic agent in the development of bioactive biomaterials for regenerative endodontics.

In addition to the previously mentioned markers, Yu et al. [11] reported that CBD was able to reverse effects produced by TNF‐*α* such as reduced viability, migration, and osteogenic/odontogenic differentiation. Their studies also reveal the attenuation of the expression of the proinflammatory cytokines Interleukin 6 (IL‐6) and Interleukin 1‐*β* (IL 1*β*). Moreover, Yu et al. [11] also identified the expression of osteocalcin and osteonectin, further supporting the osteogenic/odontogenic potential of CBD. Liu et al. [32] and Yu et al. [11] confirmed this potential by Western blot analysis, which demonstrated upregulation of proteins associated with cellular differentiation, including RUNX2, Type I collagen, BMP2, and alkaline phosphatase. The same method was employed by Qi et al. [33], who showed that cannabinoid receptors, particularly CB2, whose expression was upregulated by CBD treatment, play an important role in modulating CBD‐induced dentin regeneration by acting through the mitogen‐activated protein kinase (MAPK) signaling pathway.

Another signaling pathway investigated was the wingless‐related integration site (WNT) pathway. Liu et al. [32] linked this pathway to the enhanced osteogenic potential of both CBD‐treated microspheroids and hDPSCs through the upregulation of the wingless‐type MMTV integration site family, Member 6 (WNT6) gene cascade. Like other glycoproteins of the WNT family, this gene is directly associated with odontogenesis. mRNA expression analysis revealed a stepwise increase in WNT6 expression in the following order: hDPSCs, CBD‐treated hDPSCs, microspheroids, and CBD‐treated microspheroids. In addition, the authors evaluated the downstream WNT6 signaling pathway, specifically the *β*‐catenin axis, which is recognized as a key pathway involved in osteogenesis. *β*‐Catenin activity was experimentally confirmed through the application of Dickkopf‐related Protein 1 (DKK1), a pharmacological inhibitor of the wingless‐related integration site/beta‐catenin signaling (WNT/*β*‐catenin) pathway. The presence of DKK1 not only suppressed the activation of this signaling cascade but also reduced the protein expression of osteogenic markers such as osteocalcin and RUNX2. These findings, in conjunction with the positive effects of CBD on viability, proliferation, mineralization, and expression of specific markers, further support the central hypothesis of this scoping review.

All included studies were tested as controls with hDPSC in media with and without CBD in order to avoid the effect of the vehicle/solvent [11, 32, 33]. However, more studies are needed to discover the mechanisms of action of CBD on stem cells through the pathways of CB1, CB2, WNT6, MAPK, transient receptor potential Vanilloid‐1 (TRPV1), and peroxisome proliferator‐activated receptor gamma (PPAR‐*γ*). Beyond these signaling mechanisms, the cellular response to CBD also depends on its concentration. Across the included studies, CBD showed a clear dose‐dependent pattern in hDPSCs. Low concentrations (about 0.1–2.5 *μ*M) tended to improve cell viability, migration, and early markers of mineralization, whereas higher concentrations (around ≥ 12.5 *μ*M) were associated with reduced metabolic activity and signs of cellular stress. However, none of the studies defined an exact concentration at which CBD becomes definitively toxic, which limits the ability to establish a precise safety threshold.

Variability in CBD origin and preparation may influence the biological responses observed in hDPSCs. Among the included studies, only two, Qi et al. [33] and Liu et al. [32], explicitly reported CBD purity, both indicating values of ≥ 98%. The remaining studies did not specify purity, source (synthetic vs. plant‐derived), or formulation details. Such differences may affect CBD′s bioavailability, stability, and receptor‐binding characteristics, and could contribute to variability in outcomes related to viability, inflammatory signaling, and odontogenic differentiation. The lack of consistent reporting across studies limits the ability to determine whether observed effects are attributable to CBD itself or to differences in preparation, and therefore represents an important methodological limitation.

In summary, the studies included in this scoping review demonstrate that CBD exerts positive effects on hDPSC viability, proliferation and differentiation, angiogenesis, and expression of osteogenic/odontogenic markers, in addition to activating key biological pathways such as MAPK and WNT/*β*‐catenin. These findings reinforce the translational potential of CBD as a promising bioactive agent in regenerative endodontics and highlight the need for further clinical studies to validate its therapeutic application.

This scoping review presents some limitations. First, because it only included in vitro studies, the low number of robust clinical investigations still restricts the direct translation of these findings into clinical practice. Second, as a scoping review, it did not involve a formal risk of bias assessment or meta‐analysis, which limits the ability to critically appraise evidence strength and establish causal inferences. Future systematic reviews and clinical studies are needed to overcome these gaps.

## 5. Conclusion

CBD is a promising bioactive compound for regenerative endodontics, promoting mineralization, modulating inflammation, and enhancing key cellular events in hDPSCs. However, current evidence is limited and further studies are needed to define safe concentrations, delivery systems, and clinical applicability. Although still in its early stages, CBD research represents a novel and relevant approach in regenerative endodontics.

## Funding

This study was supported by Coordenação de Aperfeiçoamento de Pessoal de Nível Superior—Brasil (CAPES)—Finance Code 001, 001.

## Conflicts of Interest

The authors declare no conflicts of interest.

## Data Availability

Data sharing is not applicable to this article as no datasets were generated or analyzed during the current study.
